# Structural determinants of SlpA-mediated phage recognition in *Clostridioides difficile*

**DOI:** 10.1371/journal.ppat.1013724

**Published:** 2025-11-26

**Authors:** Alexia L.M. Royer, Émeline Dion, Andrew A. Umansky, Mariano Avino, Olga Soutourina, Louis-Charles Fortier

**Affiliations:** 1 Department of Microbiology and Infectious Diseases, Faculty of Medicine and Health Sciences, Université de Sherbrooke, Sherbrooke, Québec, Canada; 2 Université Paris-Saclay, CEA, CNRS, Institute for Integrative Biology of the Cell (I2BC), Gif-sur-Yvette, France; 3 Bio-informatic Platform, Department of Biochemistry and Functional Genomics, Faculty of Medicine and Health Sciences, Université de Sherbrooke, Sherbrooke, Québec, Canada; Texas A&M University, UNITED STATES OF AMERICA

## Abstract

Phage therapy is a promising alternative to antibiotics for treating *Clostridioides difficile* infections. Despite its fundamental importance, the initial and most critical step of *C. difficile* phage infection cycle - the precise recognition of a host receptor - remains poorly understood. The surface-layer protein SlpA was previously identified as a general phage receptor in *C. difficile*, but the molecular determinants of phage-S-layer interactions remain unclear. We investigated the structural features of SlpA required for phage recognition by engineering and expressing modified isoforms in FM2.5 and R20291 Δ*slpA* strains. By deleting specific SlpA domains and creating chimeric constructs from different isoforms, we assessed the capacity of seven phages to adsorb and infect the complemented strains. Our results demonstrate that both the LMW and HMW fragments of the S-layer protein contribute to phage specificity in an isoform-dependent manner. In addition, the LMW D2 domain is frequently required, but not always essential for productive infection. Several phages were also able to infect some of the complemented strains despite poor or delayed adsorption, highlighting differences in receptor binding. Interestingly, some phages adsorbed efficiently but failed to infect, showing that the capacity to bind to a host is not always predictive of a successful infection. Our findings reveal the complexity of phage-host interactions in *C. difficile* and provide new insights into the structural features of the S-layer protein that govern phage binding. These findings will be instrumental in guiding the future design of phage cocktails to target a broad spectrum of *C. difficile* clinical isolates.

## Author summary

The emergence of *Clostridioides difficile* as a leading cause of healthcare-associated nosocomial intestinal infections calls for novel therapeutic strategies, particularly in the context of antibiotic resistance and recurrent disease. Phage therapy is a promising approach, but its clinical application against *C. difficile* remains hindered by our lack of understanding of the factors driving host specificity. Indeed, it is crucial to understand how phages specifically interact with their host to be able to select the best candidates for cocktail preparation. The main surface layer protein SlpA is a key phage receptor, but the molecular details governing phage-receptor interactions remain unclear. By dissecting the structural features of the S-layer protein required for phage infection through engineered isoforms and domain modifications, we reveal how specific regions of SlpA mediate phage adsorption and infection. These new insights into phage–receptor interactions will be instrumental in guiding the future engineering of broad-host-range therapeutic phages.

## Introduction

Bacteriophages, or phages, play a central role in regulating microbial populations and shaping ecosystems through highly specific interactions with their bacterial hosts [1]. This specificity makes them powerful tools for therapeutic applications, particularly to fight against multidrug-resistant pathogens. Unlike broad-spectrum antibiotics, phages offer a targeted approach that can selectively eliminate pathogenic bacteria while preserving beneficial members of the microbiota [2].

Among the pathogens under investigation for phage therapy, *Clostridioides difficile* has gained particular interest due to its role as the leading cause of post-antibiotic gastrointestinal infections in industrialized countries [3]. *C. difficile* infections (CDI) represent a significant economic burden, with 20–25% of patients experiencing recurrence after first-line antibiotic therapy, leading to prolonged hospital stays and more expensive treatments [4,5]. Phage therapy offers a promising targeted solution, capable of reducing recurrences and hospital stays while sparing the gut microbiota [6]. Preclinical *in vivo* studies in murine and hamster models have demonstrated encouraging efficacy against CDI [7–9]. However, since all phages isolated on *C. difficile* to date have been temperate and no strictly lytic phages have been reported [3,10], the absence of such phages rapidly leads to lysogeny-mediated phage resistance. In addition, the generally narrow host range of *C. difficile* phages limits their efficacy against diverse strains, representing one major obstacle for phage therapy development [10,11].

Phage infection relies on specific interactions between phage receptor binding proteins (RBPs) and bacterial surface receptors. The specificity of these interactions, along with the presence of other antiphage systems and prophage-mediated interference, shape phage host range and modulate infection efficiency [10]. In Gram-positive bacteria, phage receptors are most often components of the cell wall, such as peptidoglycan or teichoic acids. Cell wall proteins have also been identified as receptors in few documented cases [12]. In *C. difficile*, a Gram-positive, strictly anaerobic and spore-forming bacterium, the proteinaceous surface-layer (S-layer) appears to play a key role in phage recognition. Previous studies suggested that *C. difficile* phages and phage tail-like bacteriocins (e.g., diffocins, Avidocin-CDs) use the surface-layer protein A (SlpA) as a receptor [13–17]. In our previous work, we further provided direct experimental demonstration that SlpA serves as a general receptor. This was evidenced through the isolation of a spontaneous FM2.5 mutant strain carrying a single nucleotide insertion in *slpA*. The mutation causes a frameshift and introduces a premature stop codon in the sequence, therefore abrogating SlpA production and assembly at the cell surface [13–17]. In our previous work, using the FM2.5 strain, we further provided direct experimental demonstration that the S-layer serves as a receptor for multiple *C. difficile* phages, establishing a foundation for investigating the molecular basis of phage-S-layer interactions [18].

SlpA is the main component of the *C. difficile* S-layer, a proteinaceous paracrystalline lattice covering the bacterial surface. It is synthesized and secreted as a precursor that is cleaved by the cell wall protease Cwp84 into two fragments that are reassembled at the cell surface. The high molecular weight (HMW) fragment anchors the S-layer to the cell wall and is the most conserved of the two fragments. The low molecular weight (LMW) fragment is more variable in sequence and is surface-exposed, therefore subject to strong selective pressures [19–22]. Beyond its role as a phage receptor, the S-layer contributes to virulence, surface integrity, cell adhesion and plays a role in protecting the bacterium from host immune defences [13,23–25]. Thirteen SlpA isoforms, corresponding to distinct S-Layer Cassette Types (SLCT), have been identified to date, each encoded by a variable *slpA* locus and expressed by different *C. difficile* strains [22,26]. These isoforms represent natural protein variants that differ mainly in sequence and domain composition due to cassette recombination events rather than single nucleotide polymorphisms. Structural plasticity in S-layer proteins, driven by domain swapping, insertions, and deletions, enables rapid adaptation to environmental challenges. This likely explains the diversity of S-layer isoforms in *C. difficile*, each potentially conferring a selective advantage by evading phage or immune recognition [27]. This structural and sequence plasticity is not unique to *C. difficile* and is widespread among bacteria and archaea [23,27].

S-layer proteins have been implicated as phage receptors in other bacterial species, such as the A-layer in *Aeromonas salmonicida* targeted by phage TP446 [28], and the RsaA protein in *Caulobacter crescentus* targeted by phage ΦCr30 [29]. More recent work on *Bacillus anthracis* has shown also that the S-layer protein Sap acts as a receptor for phages AP50c and γ [30]. Another particularly informative example of phage-S-layer interactions involves *Lactobacillus helveticus.* The S-layer protein SlpH was identified as the receptor for strictly lytic phages CNRZ 832-B1 and ΦLh56. This was demonstrated through the isolation of bacteriophage-insensitive mutants (BIMs) and whole-genome sequencing, which revealed point mutations and deletions in *slpH* that altered the protein’s folding and disrupted phage adsorption and infection [31–33]. To our knowledge, this remains the only study that has directly implicated specific amino acid residues of an S-layer protein in modulating phage interaction, providing molecular-level insight into this class of phage receptors.

Despite these findings, the diversity and complexity of S-layer architectures across species hinder the establishment of generalized models for phage interaction. In our previous work, we demonstrated that phages recognize several SlpA isoforms in *C. difficile*, suggesting that S-layer protein variability is a key determinant of phage host range [18]. Notably, only two morphotypes of tailed phages have been reported to infect *C. difficile* strains, namely siphophages (long, flexible and non-contractile tails) and myophages (rigid and contractile tails) [34]. Across these morphotypes, we observed a consistent trend in our previous study: siphophages (e.g., Leicestervirus) preferentially target strains expressing SLCT-4 and SLCT-11, whereas myophages (e.g., Colneyvirus, Yongloolinvirus, Sherbrookevirus) more efficiently infect SLCT-6, SLCT-8, and SLCT-10. We also investigated the role of the D2 domain of SLCT-4, which corresponds to the most exposed and variable region of the protein, located within the LMW fragment. The siphophages ΦCD38–2, ΦCD111 and ΦCD146 from our collection normally infect the wild-type (WT) strain expressing SLCT-4. Our results showed that deletion of D2 in SLCT-4 abolished both adsorption and infection by ΦCD38–2 and ΦCD146, but not by ΦCD111. These findings indicate that D2 is critical for interaction with some siphophages, yet it remained unclear whether this domain in other SLCTs also plays a role in recognition by myophages.

In general, phage adsorption is a two-step process, beginning with a reversible binding between RBPs and the bacterial surface receptor. This is followed by an irreversible interaction with the same receptor, a different one or both, that triggers DNA injection in the bacterial cell [35,36]. A recent study using ELISA and isothermal titration calorimetry showed direct interaction between the RBP from phage ΦHN10 and the LMW SlpA fragment from *C. difficile* strain HN21. The observed affinity was weak, supporting the idea that the interaction between the RBP and the S-layer protein is a reversible initial interaction [37]. Given that SlpA is part of the S-layer lattice, multiple interactions with LMW fragments may strengthen binding affinity and promote irreversible attachment. These observations raise the possibility that several parts of the S-layer or additional, yet unidentified receptors, could contribute to irreversible phage attachment leading to infection.

To clarify these phage-receptor interactions, we aimed to identify specific domains of the S-layer protein that mediate phage adsorption and infection in *C. difficile*. More specifically, we aimed to determine whether phages interact with the LMW, the HMW, or both fragments. Because all known *C. difficile* phages described to date are temperate [3,10,11,38], attempts to isolate BIMs are often confounded by the high frequency of lysogeny rather than by receptor mutations. Despite the presence of active defense mechanisms such as CRISPR-Cas and restriction–modification systems, resistance due to lysogeny represents the predominant outcome following infection under laboratory conditions [7,9,38,39]. We therefore opted for a targeted genetic strategy by engineering a collection of S-layer protein deletion mutants and chimeric variants that we expressed in two different types of *slpA*-null backgrounds. One corresponds to the previously isolated FM2.5 strain described above [13]. However, since a complete copy of *slpA* is still present in the chromosome, homologous recombination is invariably observed when closely related *slpA* alleles are introduced on plasmids for complementation. To overcome this limitation, we generated a clean R20291 Δ*slpA* mutant in which the gene was entirely deleted. By evaluating the susceptibility of these two strains to a panel of seven phages, we examined how specific regions of the S-layer protein influence phages adsorption and infection. Our approach uncovered novel interactions between phages and the S-layer and underlines the critical role of receptor targeting in the advancement of phage therapy [40].

## Results and discussion

### The LMW D2 domain is essential for phage infection in an isoform- and phage-dependent manner

In our previous work, we demonstrated that the D2 domain of the LMW fragment of the SLCT-4 is required for adsorption and infection by phages ΦCD38–2 and ΦCD146, but is dispensable for ΦCD111 [18]. These experiments were performed using a chromosomally encoded SLCT-4 ΔD2 variant constructed by Lanzoni-Mangutchi *et al.* (2022). It suggested that D2 plays a role in siphophage recognition, although its importance may vary depending on the phage. Here, we extended this analysis by including other S-layer isoforms and additional myophages to determine whether the requirement for D2 domain is a general feature, shared by both phage morphologies, or a siphophage-specific, or an isoform-specific property.

**Fig 1:**
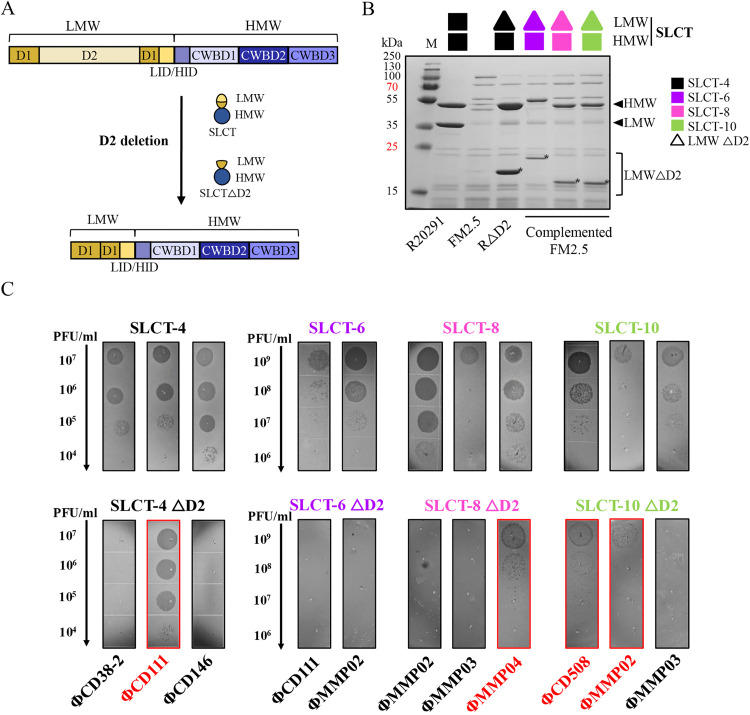
Complementation of the FM2.5 *slpA* null mutant strain with D2 deleted isoforms. A) Schematic representation of a typical S-layer cassette type (SLCT) preprotein and the effect of D2 domain deletion. The LMW fragment (yellow) comprises the D1 and D2 domains along with the LMW interacting domain (LID), while the HMW fragment (purple) contains three cell wall-binding domains (CWBD1–3) and a HMW interacting domain (HID) adjacent to LID. Deletion of the D2 domain results in an LMW fragment composed only of D1-LID. B) Coomassie-stained 12% SDS-PAGE of glycine-extracted surface proteins from WT R20291, FM2.5, the RΔD2 mutant, and FM2.5 strains complemented with D2-deleted SLCT-6, SLCT-8, or SLCT-10. Arrows indicate the major HMW and LMW fragments corresponding to SLCT-4 in the WT R20291 strain. The size of the bands varies depending on the SLCT. Asterisks (*) mark the D2-deleted LMW fragments. M = molecular weight protein marker. C) Phage susceptibility assays on the FM2.5 *slpA* mutant complemented with different D2 deleted SLCTs with siphophages (ΦCD38-2, ΦCD111, ΦCD146) and myophages (ΦCD508, ΦMMP02, ΦMMP03, ΦMMP04) that normally recognize the corresponding WT isoform. Serial 10-fold dilutions of the indicated phages (titers of undiluted phage stocks >10^8^ PFU/mL) were spotted on top of bacterial lawns. Dark zones/plaques indicate successful infection, panels showing positive results are highlighted in red. Experiments were repeated at least twice.

To test this, we complemented the FM2.5 *slpA*-null mutant strain with plasmids expressing D2-deleted versions of SLCT-6, SLCT-8, and SLCT-10 under the P_cwp2_ constitutive promoter. These SLCT variants were selected because they were known to be susceptible to infection by multiple phages [18]. The D2 domain boundaries on each SLCT were defined according to predictions by Lanzoni-Mangutchi, P. *et al.* (2022) [22] (Fig 1A). We hypothesized that, as with SLCT-4 ΔD2, deletion of D2 in other SLCTs would not impact S-layer assembly. This was confirmed by SDS-PAGE analysis of surface protein extracts, which showed the expected production and surface localization of the modified S-layer proteins (Fig 1B).

We then performed infection assays with seven phages representing siphophages (ΦCD38–2, ΦCD111, ΦCD146) and myophages (ΦCD508, ΦMMP02, ΦMMP03, ΦMMP04) that recognize at least one WT isoform, to assess the impact of the D2 deletion on susceptibility to infection. Our results showed that some myophages also needed the D2 domain for infection, depending on the SLCT (Fig 1C and S1 Table). Specifically, ΦMMP04 infected SLCT-8 ΔD2, while ΦCD508 and ΦMMP02 infected SLCT-10 ΔD2. On the contrary, ΦMMP03 was unable to infect SLCT-8 ΔD2 or SLCT-10 ΔD2. The strain expressing SLCT-6 ΔD2 was resistant to all tested phages. Notably, some phages that infected a given D2-deleted variant did not always infect all the other D2-deleted variants, despite infecting the parental WT isoform. For instance, ΦCD111 infected SLCT-4 ΔD2 but not SLCT-6 ΔD2, and ΦMMP02 infected SLCT-10 ΔD2 but not SLCT-6 ΔD2 or SLCT-8 ΔD2. These results indicate that the D2 domain of the LMW fragment is essential for binding with some siphophages and myophages but is dispensable with others and depends on the SLCT.

**Fig 2:**
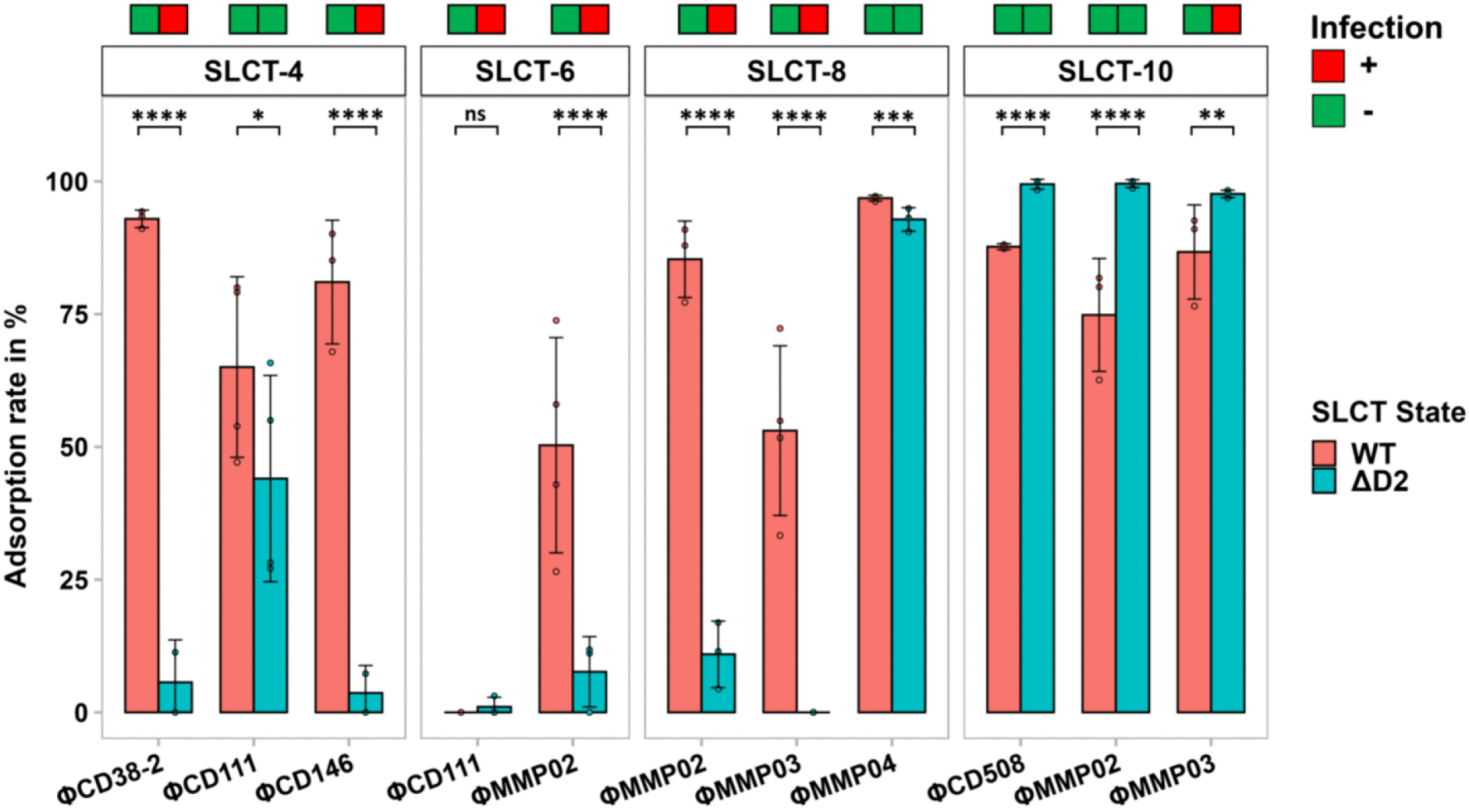
Phage adsorption assays on the FM2.5 *slpA* null mutant strains complemented with WT SLCT-4, 6, 8 and 10 isoforms and D2-deleted variants. Data presented are the mean ± SD of at least 6 technical replicates from a minimum of two independent biological experiments on different days with different cultures. *, P < 0.05, **, P < 0.01, ***, P < 0.001 and ****, P < 0.0001.

Phage adsorption tests were performed to confirm the results of infection assays. In most cases, phage infection correlated with adsorption, however exceptions were observed. For example, ΦMMP03 failed to infect the strain expressing SLCT-10 ΔD2 despite adsorbing strongly (97.7%) (Fig 2). This indicates that the D2 domain is not required for binding but may be essential for subsequent steps of the infection process. In this context, it is noteworthy to mention that adsorption generally occurs in two steps: the ﬁrst one is a reversible binding of the phage to the host cell through tail ﬁbres or other decorations, and the second step is the irreversible binding of the phage RBP to the same receptor, a different one, or both [12,41]. For instance, both the RBP (gp108) and the tail puncturing device (gp98) are necessary for *Listeria* myophage A511 to complete adsorption and initiate infection [42]. Here, we speculate that ΦMMP03 binds to the S-layer protein through its RBP but is unable to initiate infection, due to the lack of interaction with the D2 domain of the LMW fragment in the SLCT-10 ΔD2 strain. The RBP and/or another tail protein from the baseplate could be involved in this missing interaction. One thing is clear however: the absence of infection is not due to downstream interference by antiphage systems or prophages, since the strain is permissive to ΦMMP03 when a WT SLCT-10 is expressed. Further investigation on the role of phage RBP and additional tail proteins will be necessary to clarify the adsorption and infection process.

**Fig 3:**
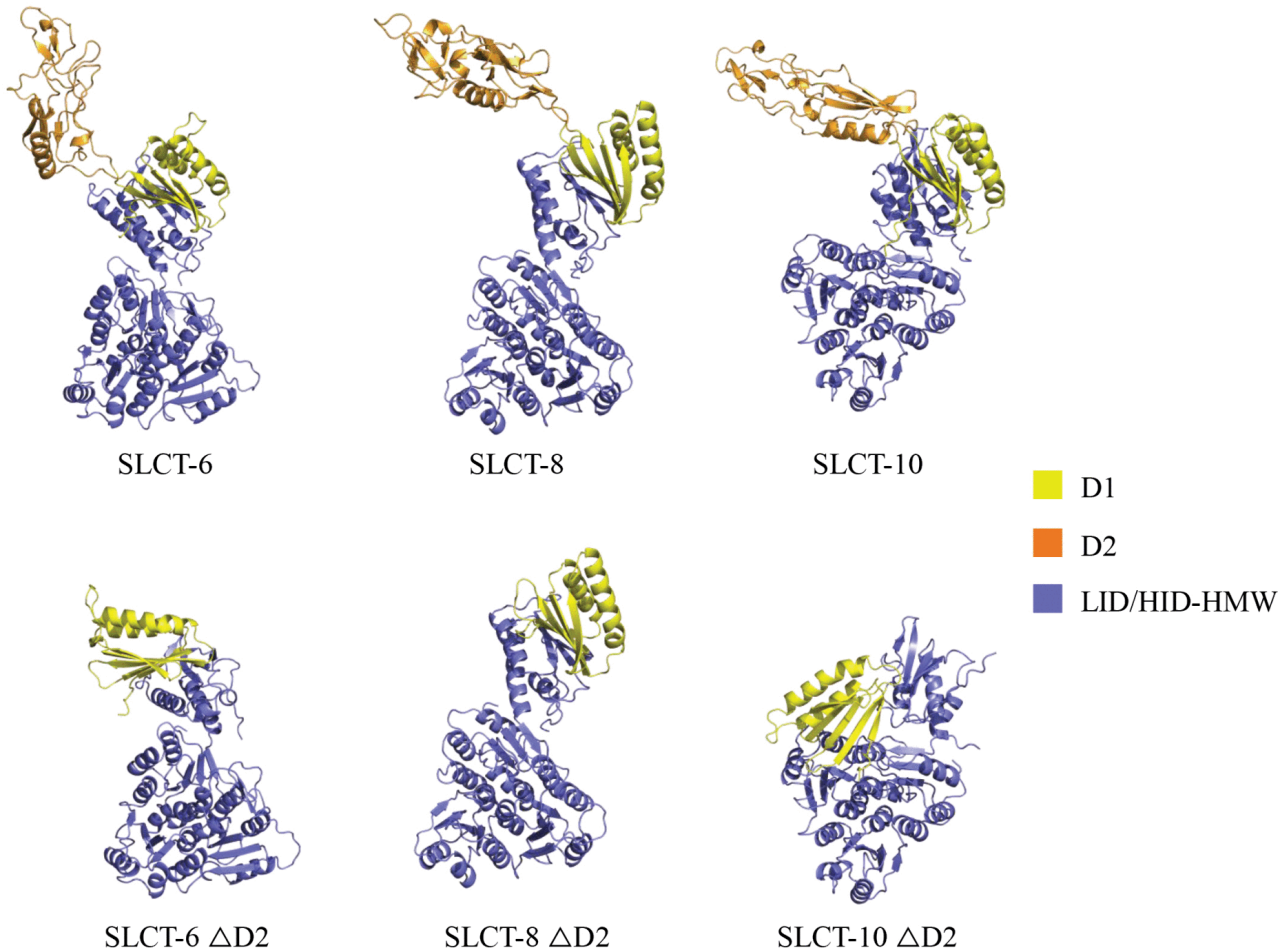
AlphaFold3 structural predictions of WT and D2-deleted SLCTs. Full-length structures (comprising both LMW and HMW fragments) of WT SLCT-6, SLCT-8, and SLCT-10, as well as their corresponding variants lacking the D2 domain, were predicted using AlphaFold3. Input sequences correspond to mature proteins (signal peptides removed), with LMW and HMW fragments submitted separately. Domains are coloured as follows: D1 in yellow, deleted D2 region in orange, and LID/HID-HMW fragment in purple. Structural models illustrate the impact of D2 deletion on overall protein folding and domain organization, in comparison with their WT counterparts.

Beyond ΦMMP03, we also observed that all phages interacting with SLCT-10 exhibited increased adsorption when D2 was deleted, suggesting that in this isoform the D2 domain may partially mask binding sites. AlphaFold3 structural predictions [43] support this hypothesis (Fig 3), as the D2-deleted SLCT-10 variant adopts a more compact conformation with a reoriented D1 domain, potentially exposing previously hidden interaction surfaces. In SLCT-6, D2 deletion also led to reorientation of the D1 and LID/HID domains, although to a lesser extent. In contrast, deletion of D2 in SLCT-8 did not result in major conformational changes according to the AlphaFold3 modelling (Fig 3). Together, these results highlight the complex and context-dependent role of the D2 domain in phage-S-layer interactions. Depending on the phage and SLCT, the D2 domain can be essential for infection, adsorption or, conversely, can hinder receptor accessibility.

**Fig 4:**
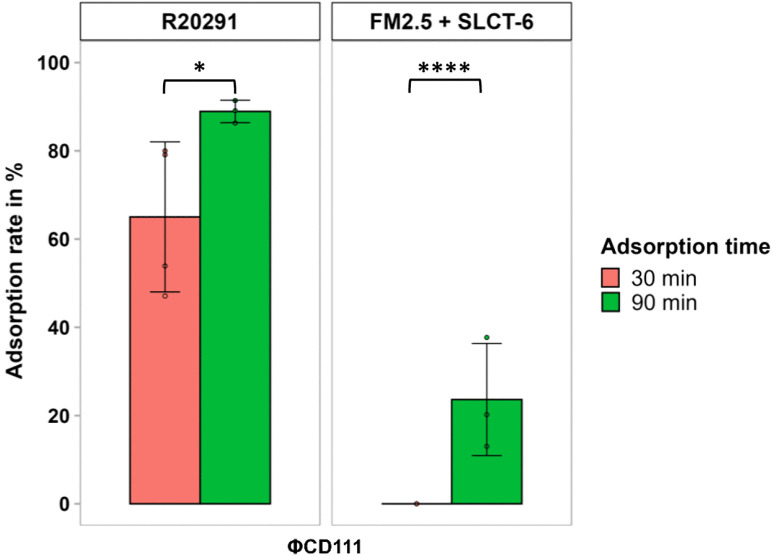
Phage adsorption time course. Adsorption time course of ΦCD111 on WT R20291 and FM2.5 complemented with SLCT-6. Data represent the mean ± SD of at least three technical replicates from a minimum of two independent biological experiments performed on different days with distinct cultures. Statistical significance was assessed using a Welch’s t-test following normality evaluation by the Shapiro–Wilk test. *, P < 0.05, and ****, P < 0,0001, ns = not significant.

Another interesting case was observed with ΦCD111 that infected SLCT-6 [18], despite showing no detectable adsorption after the standard 30-minute incubation (Fig 2 and S1 Table). To test whether these infections reflected delayed adsorption, we extended the incubation period in time-course assays. Adsorption progressively increased, reaching 23.6% after 90 minutes on SLCT-6, with a similar delayed binding pattern observed on the R20291 WT strain (Fig 4). This suggests that relying only on adsorption tests to predict phage susceptibility of a given strain is insufficient. Adsorption assays with *C. difficile* are generally done in 30 minutes [11,44]. This is clearly not enough for certain phages like ΦCD111 that require more time on certain strains. Technically, this can become challenging because once the infection is initiated, new progeny phages will be released eventually. Depending on the latent period, these newly released phages will contaminate the supernatant and invalidate the results. The characteristics of the lytic cycle of ΦCD111 have not been determined, but in a previous study we have determined that ΦCD38–2, which is genetically very similar to ΦCD111, has a latent period of 95 minutes [45]. Therefore, we could extend our adsorption assays up to 120 minutes without observing new phages in the culture supernatant. In fact, delayed adsorption might be more frequent than expected, as a similar case was previously reported for phage ΦCDHM6, which exhibited less than 20% adsorption on its propagation host [44]. Consequently, low adsorption does not always prevent successful infection and underscores the importance of combining adsorption and infection assays to fully capture phage-host interaction dynamics.

### Construction of a Δs*lpA* R20291 strain for studying phage–receptor interactions

Studying phage–receptor interactions requires a stable *slpA* null background. The FM2.5 mutant, which contains a premature stop codon in *slpA*, is prone to homologous recombination restoring the WT allele when complemented with SLCT-4. [13]. This event likely results from the presence of a complete but non-functional *slpA* sequence in the FM2.5 chromosome, which may facilitate homologous recombination and reflect a strong selective pressure for S-layer restoration. To circumvent this, we generated a complete *slpA* deletion (Δ*slpA*) in the epidemic strain R20291 using the endogenous CRISPR-Cas system [40]. A similar mutant has been recently reported in the CD630 strain and a CRISPR interference (CRISPRi)-mediated knockdown was achieved in the R20291 strain [46,47].

**Fig 5:**
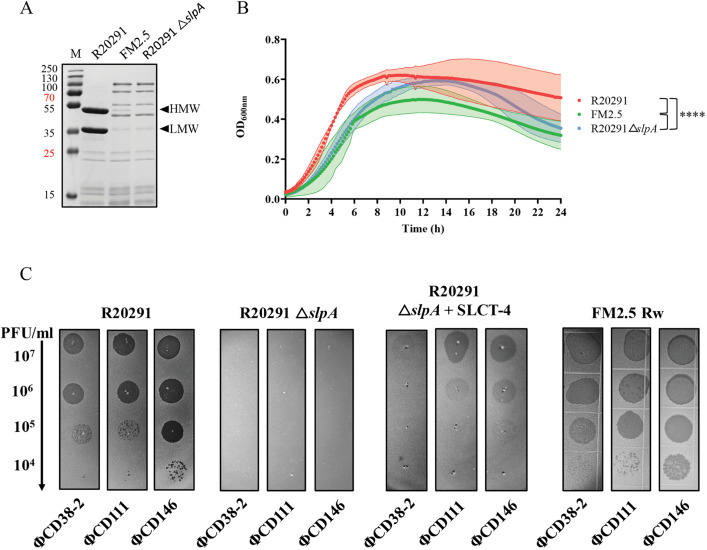
Characterization of the *slpA* deletion in *C. difficile* R20291 and its impact on surface protein profile, growth, and phage susceptibility. A) Coomassie-stained 12% SDS-PAGE of glycine-extracted surface proteins from R20291 WT, FM2.5, and R20291 Δ*slpA* strains. Arrows indicate the two major bands corresponding to the HMW and LMW fragments of SLCT-4, naturally present in R20291 and absent in FM2.5 and Δ*slpA* strains. M = molecular weight protein marker. B) Growth curves of R20291 WT (red), FM2.5 (green), and R20291 Δ*slpA* (blue) in liquid culture over 24 h. Curves represent the mean of biological triplicates; shaded areas indicate standard deviation. Statistical analysis: two-way ANOVA with time and strain as factors and Tukey’s post hoc test on marginal means. Significance: **** = p < 0.0001. C) Susceptibility to phages ΦCD38-2, ΦCD111 and ΦCD146 using spot tests. Serial 10-fold dilutions of each phage (titers of undiluted phage stocks >10^8^ PFU/mL) were spotted on lawns of R20291 WT, R20291 Δ*slpA*, and R20291 Δ*slpA* complemented with a plasmid expressing SLCT-4. The assay performed on R20291 strain is the same as the one shown in Fig 1C. Dark zones indicate bacterial lysis and susceptibility to infection.

To verify that the deletion of *slpA* effectively abolished S-layer formation and to evaluate potential physiological consequences, we analyzed cell wall protein profiles and growth of the R20291 Δ*slpA* mutant in comparison with the FM2.5 and WT strains. SDS-PAGE analysis of cell wall protein extracts confirmed the absence of the S-layer protein on the cell surface, and the presence of protein patterns similar to those found with the FM2.5 strain (Fig 5A). Growth curve analysis showed that the FM2.5 and R20291 Δ*slpA* strains exhibited similar exponential growth, both reduced compared to the WT R20291 strain. The R20291 Δ*slpA* strain also reached a slightly higher maximum optical density at 600 nm (OD_600_) during the late exponential phase, similar to the WT strain (Fig 5B). Statistical analysis was performed using a two-way ANOVA with time and strain as factors, revealing significant effects of strain (p < 0.0001) and time (p < 0.0001), with no interaction between the two factors (p = 0.9996). Tukey’s post hoc test on marginal means showed that R20291 differed significantly from both FM2.5 and Δ*slpA* (p < 0.0001), and that FM2.5 and Δ*slpA* also differed significantly (p < 0.0001). These results indicate that the three growth curves are statistically distinct. Both mutant strains showed a decline in OD₆₀₀ during stationary phase, consistent with previous reports describing an increased tendency for autolysis in FM2.5 [13,46]. Although we did not directly measure autolysis, our observations align with these previous findings. Despite their similar trends, statistical analysis confirmed that FM2.5 and R20291 Δ*slpA* differed significantly from each other, as well as from the WT strain, indicating that disruption of *slpA*, either by point mutation or deletion, seems to affect growth dynamics differently.

**Fig 6:**
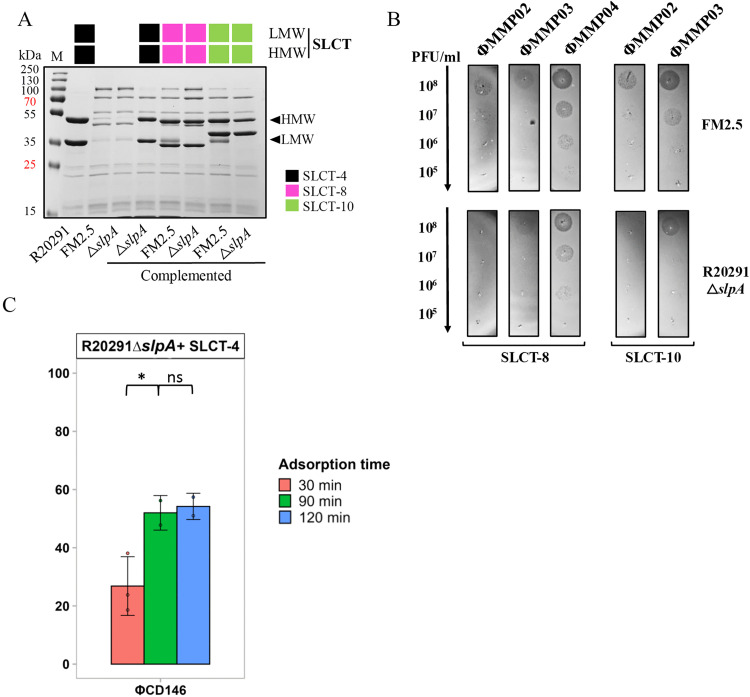
Complementation of *C. difficile* R20291 Δ*slpA* with different SLCT. A) Coomassie-stained 12% SDS-PAGE of glycine-extracted surface proteins from WT R20291, FM2.5, R20291 Δ*slpA*, and both FM2.5 and R20291 Δ*slpA* strains complemented with SLCT-4, SLCT-8, or SLCT-10. Arrows indicate the HMW and LMW fragments corresponding to the SLCT-4 S-layer naturally present in the R20291 strain. M = molecular weight protein marker. B) Spot test comparing myophage susceptibility of FM2.5 and R20291 Δ*slpA* strains complemented with SLCT-8 or SLCT-10. Serial 10-fold dilutions of phages ΦMMP02, ΦMMP03, and ΦMMP04 (titers >10⁸ PFU/mL) were spotted onto bacterial lawns of the indicated strains. Dark zones indicate bacterial lysis and susceptibility to infection. C) Adsorption time course of ΦCD146 on strain R20291 Δ*slpA* complemented with SLCT-4. Data represent the mean ± SD of at least three technical replicates from a minimum of two independent biological experiments performed on different days with distinct cultures. Statistical significance was assessed using a Welch’s t-test following normality evaluation by the Shapiro–Wilk test. *, P < 0.05, and ****, P < 0,0001, ns = not significant.

To confirm that deletion of *slpA* conferred complete resistance to phage infection, as observed with FM2.5, and to assess whether complementation with *slpA* restored susceptibility, we performed infection assays using the three siphophages infecting the R20291 WT strain. As previously shown, the R20291 strain produced clear, well-defined plaques upon infection with the three siphophages tested (ΦCD38–2, ΦCD111, and ΦCD146) [18], whereas the R20291 Δ*slpA* mutant was completely resistant, showing no lysis, consistent with the phenotype previously observed for FM2.5 in our earlier study (Fig 5C) [18]. Complementation of the R20291 Δ*slpA* mutant with plasmid-borne SLCT-4 partially restored susceptibility, resulting in turbid lysis zones rather than discrete plaques. For ΦCD38–2, only a single turbid lysis zone was visible under these conditions (Fig 5C), although multiple plaques were observed in full soft agar overlays, suggesting partial restoration of infectivity. Because plasmid-borne expression of SLCT-4 in FM2.5 leads to immediate homologous recombination with the chromosomal copy of *slpA*, we used the revertant strain FM2.5 Rw instead, in which the native watermarked *slpA* sequence was restored by spontaneous recombination [13]. As expected, FM2.5 Rw recovered full phage sensitivity comparable to the WT R20291 strain (Fig 5C) [18]. Next, we complemented the R20291 Δ*slpA* strain with different WT SLCTs (SLCT-8, and SLCT-10) and compared them to the corresponding FM2.5 complemented strains. SDS-PAGE confirmed the detection of comparable levels of S-layer protein in both genetic backgrounds (Fig 6A). However, phage infection assays revealed reduced infection efficiency in the R20291 Δ*slpA* background. The complemented R20291 Δ*slpA* strains displayed fewer plaques or faint lysis zones across phage dilutions (Fig 6B). For example, ΦMMP02 failed to form plaques on SLCT-8 and SLCT-10 complemented R20291 *ΔslpA*, while still producing plaques in the FM2.5 background. Likewise, the efficiency of plaquing (EOP) for ΦCD38–2, ΦCD111 and ΦCD146 on SLCT-4 complemented R20291 Δ*slpA* strain was 10^-2^ compared to the R20291 WT strain (S1 Fig).

To investigate this discrepancy, phage adsorption assays were performed and revealed a consistent delay in phage binding in the R20291 Δ*slpA* background relative to R20291 with phage ΦCD146 reaching 26.8% adsorption after 30 min of incubation but 54.2% after 90 min (Fig 6C). This suggests surface-related differences or receptor presentation affecting phage accessibility. Previous studies in *C. difficile* FM2.5, FM2.5 variants (FM2.5varA and FM2.5varB carrying point mutations restoring the *slpA* reading frame and translation) and CD630 *ΔermΔslpA* have shown that the absence of S-layer protein, or even a modified structural organization of the S-layer, as observed with FM2.5varB, alters the surface composition, with up- and downregulation of other cell wall proteins (Cwps), including upregulation of CwpV [39,46,48]. Consistently, SDS–PAGE revealed additional bands at ~100 kDa and ~42 kDa in our R20291 Δ*slpA* strain, likely corresponding to CwpV fragments. CwpV has been shown to promote cell aggregation when overexpressed [49], and we have also previously shown that it confers resistance against phages in the R20291 strain, particularly against siphophages [39]. However, these additional Cwps are also present in the FM2.5 mutant, so there is likely another explanation for the noted differences in terms of phage infectivity.

Whole-genome sequencing was carried out to detect possible background mutations in the R20291 ∆*slpA* strain that could explain the observed discrepancies. Sequencing confirmed the complete deletion of *slpA*. Comparative genome analysis of the parental R20291 strain, the R20291 Δ*slpA* mutant, and FM2.5 strains revealed three missense mutations unique to the R20291 ∆*slpA* strain (S3 Table). The mutations were found in genes encoding a tyrosine recombinase (*xerD2*), a putative transcriptional antiterminator, and a predicted CAAX amino terminal protease. InterProScan analysis indicated that the *xerD2* mutation corresponds to an aspartic acid to tyrosine substitution (D/Y) located between the predicted DNA-binding domain and the catalytic core of the protein. The antiterminator mutation also involves a D/Y substitution, although it lies within a region not assigned to a defined protein domain. For the CAAX amino terminal protease, the mutation results in a serine to proline change (S/P) within a cytoplasmic domain of unknown function. The role of these mutations in the observed phenotypes is unknown and will require further investigation. In contrast, the FM2.5 strain carried only one nucleotide insertion creating a premature stop codon in the *slpA* gene, as previously described [13] (S4 Table). Five other single nucleotide polymorphisms (SNPs) were shared between our WT R20291 isolate, the R20291 ∆*slpA* mutant, and FM2.5, but not found in the published R20291 reference genome (NC_013316), indicating divergence from the reference strain (S5 Table). Collectively, whole genome sequencing revealed background mutations in the R20291 Δ*slpA* mutant compared with FM2.5. While such mutations could potentially reflect adaptive processes associated with the absence of S-layer protein, further work with independently constructed mutants would be required to determine whether they are reproducible adaptive events or construction-associated background neutral mutations. Considering the observed reduction in phage infectivity in the R20291 Δ*slpA* strain, we restricted our use of this strain to complementation assays with SLCT-4 variants that cannot be otherwise stably expressed in FM2.5 due to recombination events.

### Complete deletion of the LMW fragment leads to phage resistance

**Fig 7:**
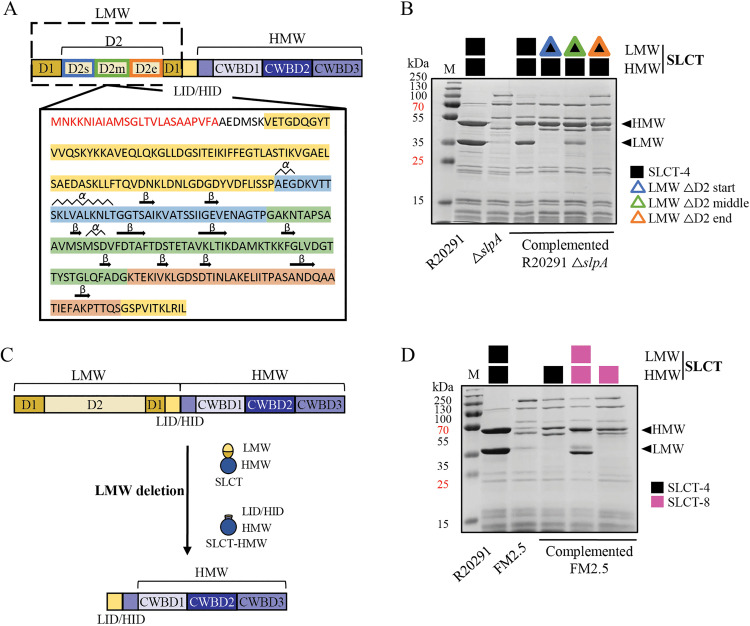
Expression of partial D2 deletions and HMW domain of SLCT-4 in *slpA* null mutant strains. A) Schematic representation of SLCT-4 preproteins with the boundaries of the D2 subdomains for the construction of three partial deletion variants. Color code: red amino acids = signal peptide, yellow = D1 domain, blue = D2 start, green = D2 middle and orange = D2 end subdomains. Secondary structures within the D2 domain, shown as arrows (β-sheets) and waves (α-helices), were predicted based on the crystallographic structure of SLCT-7 from the *C. difficile* CD630 strain (Lanzoni-Mangutchi *et al.*, 2022). B) Coomassie-stained 12% SDS-PAGE of glycine-extracted surface proteins from WT R20291, FM2.5, R20291 Δ*slpA*, and R20291 Δ*slpA* strains complemented with plasmids encoding WT SLCT-4 or SLCT-4 variants carrying partial D2 deletions, which express only the HMW-4 fragment. C) Schematic representation of HMW SLCT-4 preprotein with LID. The LID domain is shown in yellow and the HMW fragment in purple. D) Coomassie-stained 12% SDS-PAGE of surface protein extracts from WT R20291, FM2.5, and FM2.5 strains complemented with SLCT-H4, SLCT-8, or SLCT-H8. Arrows indicate the HMW and LMW fragments corresponding to SLCT-4 in R20291. M = molecular weight protein marker.

Having established a stable R20291 Δ*slpA* background that allowed expression of SLCT-4 variants without recombination problems, we next examined the contribution of the LMW fragment in phage receptor function, and particularly D2 subdomains. As previously shown, the D2 domain of SLCT-4 is essential for infection by siphophages ΦCD38–2 and ΦCD146, but not by ΦCD111 [18]. The phage- and isoform-specific D2 requirement, as seen in Fig 1C, suggests that certain structural elements within D2 may act as recognition sites. To refine our understanding of which D2 subdomains may mediate phage interaction, we designed three internal deletions based on previous secondary structure predictions [22]. Specifically, we targeted regions containing clusters of β-sheets and α-helices within the LMW D2 domain, generating one deletion per strain (Fig 7A). To assess the effect of these internal deletions, we first validated the production of the S-layer protein on the cell surface. SDS-PAGE analysis revealed the absence of the LMW fragment when partial D2 deletions were introduced, while the HMW fragment could be detected (Fig 7B). An unrelated band comigrating with the LMW fragment can be observed in the lane corresponding to the LMW ∆D2 middle deletion mutant, but a similar band has been observed in FM2.5 strains complemented with SLCT-8 and SLCT-10 (Fig 7D). Similar deletions in other S-layer isoforms have previously been reported to weaken the interaction between the LMW and HMW fragments, resulting in the loss of S-layer protein from the bacterial surface and its release into the supernatant [50]. Here, the HMW fragment could still be detected on the cell surface, which to our knowledge is the first instance of a *C. difficile* strain expressing only the HMW fragment.

Phage susceptibility assays revealed that none of the tested phages could infect the modified strains with the partial D2 deletions that turned out to express only the SLCT-4 HMW fragment (S2 Fig). To further confirm whether the HMW fragment alone could support or not phage infection, we engineered a new strain expressing only the HMW portion of SLCT-4. This construct was generated by deleting both D1 and D2 regions from the LMW fragment while retaining the LID and HID domains, which are necessary for correct processing and reassembly of S-layer proteins (Fig 7C) [22]. Retention of these motifs was intended to preserve the overall integrity of the HMW fragment on the surface.

As expected, this strain expressed only the HMW fragment when surface proteins were analysed by SDS-PAGE (Fig 7D). However, it did not support phage infection for all tested phages, and only three phages showed detectable adsorption (S4A Fig and S1 Table). Among them, ΦCD38–2 and ΦCD146, which normally infect and adsorb to SCLT-4 but not SLCT-4 ∆D2, exhibited ~30% and ~37% adsorption, underpinning the essentiality of the LMW fragment - or part of it - for phage binding and infection. Notably, ΦMMP03, which neither infects nor adsorbs to the WT SLCT-4, showed ~35% adsorption to cells expressing only the HMW. While the significance of these interactions remains unclear, it suggests that ΦMMP03 may weakly engage interaction with the HMW fragment in the absence of the LMW domain, although this interaction is insufficient to support productive infection.

Collectively, our findings suggest that for all the phages tested, some regions of the LMW are essential for infection and the D2 domain is sometimes dispensable. While complete deletion of the D2 domain does not affect the surface production of the LMW fragment, partial D2 deletions destabilize the LMW-HMW complex reassembly, resulting in the loss of the LMW fragment from the cell surface, and impaired phage receptor function. This phenotype likely reflects local structural misfolding, as supported by AlphaFold3 predictions, showing increased structural flexibility and disorder with D2 reorientation in the LMW region (S3 Fig). This suggests that the LMW portions may fail to assemble properly or are degraded due to structural defects. These flexible regions may destabilize the protein, prevent proper cleavage by the Cwp84 protease and/or impair fragment reassembly to the cell wall. This is consistent with structural data from Ormsby *et al*. (2023) who showed that disruptions in α2L helice in D1 and adjacent loops can destabilize D1-D1 interactions and alter the global organization of the *C. difficile* S-layer lattice [48]. Similarly, Lanzoni-Mangutchi *et al.* (2022) demonstrated that point mutations in LID (F274A) or HID (Y27A) domains destabilize the H/L complex, causing shedding of LMW and truncation of HMW [22]. Together, these findings underline the critical structural roles of D2, LID, and other flexible regions in S-layer protein architecture. Identified as highly dynamic in both crystallographic models and our AlphaFold3 predictions, these domains likely act as structural hinges. Their removal or alteration may perturb interdomain interactions essential for S-layer assembly and for exposing or stabilizing phage-binding surfaces. Taken together, these results indicate that loss or structural disruption of the LMW fragment abolishes phage infection, confirming that this region constitutes the core functional determinant of S-layer protein-mediated phage recognition in *C. difficile*. Finally, to our knowledge, this is the first report of a *C. difficile* strain expressing only the HMW fragment of the S-layer, providing a unique tool to further investigate S-layer protein assembly and the molecular basis of phage–receptor interactions.

### Chimeric SlpA isoforms reveal fragment-specific contributions to phage recognition

**Fig 8:**
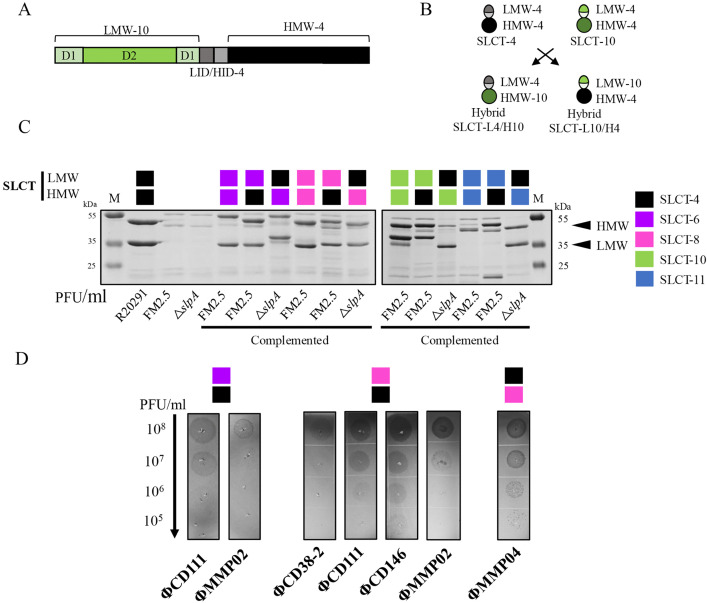
Construction and expression of chimeric SLCTs. A) Schematic representation of a chimeric SLCT preprotein composed of LMW domains D1 and D2 from SLCT-10 (green) and the LID/HID-HMW portion from SLCT-4 (black). B) Design of domain-swapped chimeric SLCTs generated by exchanging D1 and D2 domains between SLCT-4 and SLCT-10, resulting in constructs SLCT-L4/H10 and SLCT-L10/H4. C) Coomassie-stained 12% SDS-PAGE of glycine-extracted surface proteins from WT R20291, FM2.5, R20291 Δ*slpA*, FM2.5 strains complemented with native SLCTs (SLCT-6, SLCT-8, SLCT-10, SLCT-11), and *slpA* null strains (FM2.5 or R20291 Δ*slpA*) complemented with chimeric SLCTs (SLCT-L6/H4, SLCT-L4/H6, SLCT-L8/H4, SLCT-L4/H8, SLCT-L10/H4, SLCT-L4/H10, SLCT-L11/H4, SLCT-L4/H11). Arrows indicate the HMW and LMW fragments of SLCT-4, naturally present in R20291. Band sizes vary by SLCT. M = molecular weight protein marker. D) Spot test showing phage susceptibility of strains complemented with chimeric SLCTs. Positive infections were observed for FM2.5 + SLCT-L6/H4, R20291 Δ*slpA* + SLCT-L4/H8, and FM2.5 + SLCT-L8/H4. Serial 10-fold dilutions of siphophages (ΦCD38-2, ΦCD111, ΦCD146) and myophages (ΦMMP02, ΦMMP04) were spotted on lawns of the indicated strains (titers of undiluted phage stocks >10^8^ PFU/mL). Dark zones or plaques indicate bacterial lysis and susceptibility to infection.

To further investigate whether phages interact with the LMW fragment only (in particular the D1 domain) or both LMW and HMW fragments, we engineered eight chimeric SLCTs by exchanging the D1 and D2 domains between different SLCTs while preserving the LID and HID domains from the LMW and HMW fragments to ensure proper proteolytic cleavage by the Cwp84 protease and reassembly of the protein (Fig 8A and 8B). Cleavage sites between LID/HID were determined based on previous predictions of maturation cleavage sites within S-layer proteins from 14 *C. difficile* ribotypes [51]. Our constructs included combinations of SLCT-4 with SLCT-6, SLCT-8, SLCT-10, and SLCT-11. Chimeras with the LMW fragment from SLCT-6, SLCT-8, SLCT-10 or SLCT-11 fused to HMW-4 were introduced into the FM2.5 strain. However, to avoid recombination events with LMW-4 in FM2.5, the reciprocal chimeras consisting of the HMW fragment from SLCT-6, SLCT-8, SLCT-10 or SLCT-11 fused to LMW-4 were expressed in the R20291 Δ*slpA* strain. Detection of the chimeric S-layer variant proteins was confirmed by SDS-PAGE following surface protein extraction, ruling out major defects in assembly or secretion prior to phage susceptibility testing (Fig 8C). Interestingly, the LMW fragment from SLCT-11 is normally glycosylated and the size of the protein band observed on SDS-PAGE (~42 KDa) is larger than the expected ~20 KDa. In *C. difficile*, the LMW fragment of SLCT-11 is known to lack D2 domain and to be O-glycosylated within D1 at threonine 38, which causes its apparent migration at ~42 kDa on SDS-PAGE, well above its predicted unglycosylated mass (~20 kDa). In our SLCT-L11/H4 chimera, threonine 38 was preserved in D1 from SLCT-11, but a sharp band at ~20 kDa was observed on gel, consistent with loss of the glycan. We hypothesize that replacing the SLCT-11 LID with that of SLCT-4 in our chimera occluded the native threonine 38 that serves as O-glycosylation acceptors in SLCT-11. It could also have altered local conformation sufficiently to prevent proper glycan assembly. Further targeted structural and biochemical mapping will be required to clarify this [52].

Phage infection assays revealed that, in most cases, both LMW and HMW fragments from the same S-layer isoform were required for phage infection. However, some phages successfully infected certain chimeric SLCTs, indicating that they can tolerate hybrid S-layer protein architectures. For instance, both ΦCD111 and ΦMMP02 infected SLCT-L6/H4, while ΦCD38–2, ΦCD111, ΦCD146 and ΦMMP02 infected SLCT-L8/H4. Likewise, ΦMMP04 specifically targeted SLCT-L4/H8 (Fig 8D and Tables 1 and S1). Notably, ΦCD38–2, ΦCD111 and ΦCD146 phages produced diffuse and turbid lysis zones on the SLCT-L8/H4 strain, especially in the case of ΦCD38–2 infection, where individual plaques were difficult to distinguish. These altered lysis phenotypes might result from both suboptimal interaction with chimeric S-layer. Notably, the three siphophages were able to infect the SLCT-L8/H4 strain despite undetectable adsorption after the standard 30-minute incubation, suggesting that binding is weak and requires more time to stabilize. In contrast, ΦMMP03 adsorbed to both SLCT-6 (80.5%) and SLCT-L6/H4 (59.6%) but failed to infect the corresponding strains (Table 1 and S5 Fig). Since ΦMMP03 can infect the FM2.5 strain when an appropriate receptor is provided (e.g., SLCT-10) (Fig 1C), this result implies that another interaction is required to trigger the infection. These findings suggest that certain phages recognize conserved motifs or structural features within the LMW and/or HMW fragments even in non-native SLCTs combinations.

**Table 1:** Susceptibility to phage infection and phage adsorption on the FM2.5 or R20291 △*slpA* complemented strains with various SLCTs.

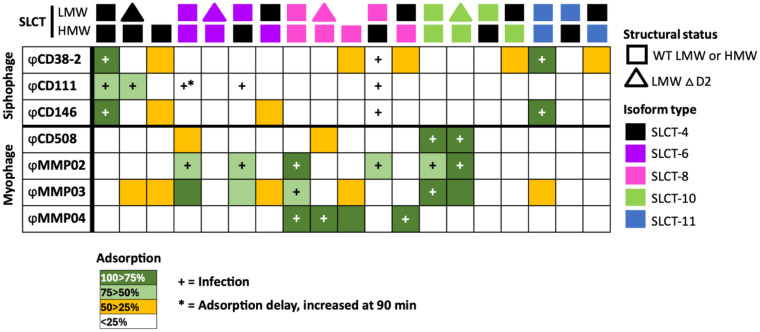

Overall, our findings suggest that phage attachment to S-layer proteins does not rely on high-affinity binding to a single, well-defined site, but instead may involve distributed, multivalent interactions across the S-layer lattice. This is consistent with the weak binding observed between the *C. difficile* phage ΦHN10 RBP and the LMW fragment of S-layer protein [37]. In contrast to classical high-affinity protein receptors such as FhuA in *Escherichia coli* [53], which are present in low copy numbers and can trigger infection through a single strong interaction, S-layer proteins are abundant, repetitive, and structurally variable. This situation more closely resembles phage interactions with carbohydrate-based receptors like LPS or wall teichoic acids, where multivalent binding helps compensate for the lower affinity of individual contacts [12,54]. In the case of S-layer proteins, the organization of RBPs in trimers or other multimeric structures on the phage tail may further enhance binding efficiency by simultaneously engaging multiple S-layer protein units [17,55]. This unique combination of a proteinaceous, abundant receptor and weak per-site affinity highlights the need to study phage-S-layer interactions as a distinct mechanistic class.

Typical phage-host studies generally involve the use of multiple phages against various bacterial strains. One major limitation of this approach is the presence of diverse factors that can interfere with phage infection. For instance, multiple antiphage systems have been predicted in *C. difficile*, among which some have been experimentally validated, like the type I-B CRISPR-Cas system and restriction – modification systems [56,57]. Prophages can also confer superinfection exclusion or CI-mediated immunity [58]. Here, by using a single genetic background to express different phage receptors to test multiple phage-host interactions, we were able to eliminate most of these common hurdles. However, our study also has some limitations. For example, the abundance of the different SLCTs detected at the surface is generally lower than what can be observed in WT strains expressing S-layer protein from the chromosome. Also, even if the predicted protein fragments were observed in cell-surface extractions, we cannot rule out the possibility that some SLCT variant could be misfolded on the cell surface, thereby impacting phage adsorption. We and others have observed that *slpA* mutant cells tend to compensate for the lack of S-layer by overexpressing other Cwps [13,46,49]. The impact of these Cwps needs to be further investigated, since some of them could interfere with phage adsorption and/or infection, like CwpV [39].

It is important to note that the lack of strictly lytic phages in *C. difficile* makes the isolation of spontaneous BIMs challenging. In fact, most of the phage resistance colonies that can be isolated after an infection are lysogens. Trying to identify spontaneous mutants of the S-layer protein to investigate receptor recognition is therefore technically very difficult. The FM2.5 mutant, which carries a point mutation, was isolated using diffocins, which are lytic tail puncturing nano machines that cannot lead to lysogeny [13]. However, with temperate phages, lysogens will invariably be isolated most of the time. The availability of strictly lytic phage variants will eventually make it possible to use phages as a selective pressure to isolate additional S-layer protein mutants and refine our understanding of phage-host interactions in *C. difficile* [8]. In the meantime, the strategy described in this work is the best at the moment to explore this important phenomenon.

### Proposed scenarios of interaction between *C. difficile* phages and the S-layer

Comparison of all the infection and adsorption patterns across all engineered SLCTs, including chimeric and domain-deleted variants, allowed us to delineate three main scenarios of interaction, each involving different combinations of structural determinants within the LMW and HMW fragments.

The first scenario involves phage interaction with both LMW and HMW fragments from natural S-layer isoforms. This is best exemplified by ΦCD146, which infects strains expressing intact SLCT-4 or SLCT-11, but not D2-deleted variants (Table 1). An exception was observed however with the SLCT-L8/H4 chimera, which ΦCD146 recognized despite being unable to infect strains expressing the natural SLCT-8 (Fig 8D and Tables 1 and S1). This suggests that the phage primarily engages with HMW-4 but also benefits from structural elements within LMW-8, likely within the D2 domain, to initiate infection. The inability of ΦCD146 to infect strains expressing only HMW-4, despite partial adsorption (30.4%) (Tables 1 and S1 and S4A Fig), supports a model in which productive infection requires engagement with both LMW and HMW fragments, either simultaneously or sequentially.

The second scenario primarily involves interaction with the LMW fragment and is exemplified by ΦMMP02. The infection pattern of this phage depends on the presence of an intact LMW from a permissive SLCT, regardless of the associated HMW fragment, except for SLCT-10 (Table 1). For example, ΦMMP02 infected SLCT-L8/H4 but not the reciprocal SLCT-L4/H8 chimera, indicating that recognition depends mainly on LMW-8 (Figs 8D and S6 and Tables 1 and S1). Deletion of LMW-8, or specifically its D2 domain, abolished infection, confirming that D2 contributes directly to phage recognition (Figs 1C and S4B and Tables 1 and S1). A similar dependence on D2 was observed with SLCT-6 and its D2-deleted variant. These results indicate that ΦMMP02 primarily targets the LMW fragment, most often within D2, but that the precise domain involved can differ across SLCTs, reflecting a flexible recognition mechanism.

A third scenario would involve interactions with the HMW and D1 domains and is exemplified by ΦMMP04. This phage was able to infect both SLCT-8 ΔD2 mutant and SLCT-L4/H8 chimera (Tables 1 and S1 and Figs 1 and 8D), suggesting recognition of the HMW-8 domain. To further test this hypothesis, we constructed a FM2.5 strain expressing only the HMW-8 fragment on a plasmid, using the same strategy as previously described for HMW-4 (Fig 7C and 7D). ΦMMP04 adsorbed efficiently (99.8%) to the SLCT-H8 strain but failed to form plaques, indicating that adsorption to HMW-8 alone is insufficient for productive infection. ΦMMP04 also bound weakly to SLCT-H4 (24.6% adsorption), indicating that it likely recognizes structural or sequence-specific features unique to SLCT-8, while conserved elements within D1 are required for infection. Together, these observations indicate that ΦMMP04 binds the HMW-8 fragment and requires the D1 domain to initiate infection. A similar observation was made with ΦMMP02 on SLCT-10: the phage retained its ability to infect SLCT-10 ΔD2, but not SLCT-4/SLCT-10 reciprocal chimeras, suggesting that this phage could possibly require the D1 domain from SLCT-10 and the associated HMW fragment.

In addition, ΦMMP04 occasionally produced faint lysis zones on other strains, including SLCT-H4, despite the absence of adsorption and infection (Tables 1 and S1 and S4B Fig). This phenomenon is commonly observed with undiluted or minimally diluted lysates of this phage. Indeed, ΦMMP04 encodes tail-associated proteins with predicted hydrolytic activity, suggesting that the observed lysis may result from a “lysis from without” phenomenon rather than productive infection [59,60].This phenomenon is therefore considered a non-specific lytic effect unrelated to phage adsorption or host recognition. A similar but much weaker phenotype was sporadically observed with other phages. For instance, ΦCD146 produced faint lysis on strains expressing SLCT-L4/H6 and L4/H8 chimeras, despite the absence of detectable adsorption and plaque formation, which could be attributed to the R20291 Δ*slpA* background (S5 and S6 Figs). Likewise, ΦMMP03 and ΦMMP02 occasionally generated a single lysis zone on certain chimeric strains (S5, S6, S7 and S8 Figs), but the lack of visible plaques and adsorption suggests that these events do not reflect productive infection and likely result from non-specific or unstable interactions.

In conclusion, our study has uncovered important aspects of the interaction between phages and the S-layer in *C. difficile*. Further investigation with strictly lytic phages and RBP-engineered phages will allow refining our understanding of the initial steps of phage infection [8,61]. Altogether, these data will be highly valuable to guide the future genetic engineering of *C. difficile* phages for therapeutic applications.

## Materials and methods

### Bacterial strains, plasmids, and bacteriophages

*C. difficile* was grown in an anaerobic chamber (Coy laboratories or Jacomex) filled with 5% or 10% H_2_, 5% CO_2_, and the balance with N_2_ at 37°C in pre-reduced TY broth (3% tryptose or tryptone, 2% yeast extract, pH 7.4). Thiamphenicol (15 µg/mL) was added for strains carrying plasmids and norfloxacin (12 µg/mL), cycloserine (250 µg/mL) or cefoxitin (8 µg/mL) was added when necessary to select *C. difficile*. *E. coli* was grown under aerobic conditions in Luria-Bertani (LB) broth at 37°C with agitation. Chloramphenicol (25 µg/mL), kanamycin (50 µg/mL) or ampicillin (50 µg/mL) was added when necessary for plasmid maintenance. All bacterial strains used in this work are presented in Table 2.

**Table 2:** List of strains, plasmids, and phages used in this study.

Strain, plasmid or phage	Characteristics	Reference
*Escherichia coli* strains		
CA434		[62]
NEBexpress		New England Biolab
HB101		Laboratory stock
LCUS 1388	CA434 + pAR01	This study
LCUS 1495	HB101 + pAR03	This study
LCUS 1497	HB101 + pAR05.2	This study
LCUS 1498	HB101 + pAR04.2	This study
LCUS 1499	HB101 + pAR07.2	This study
LCUS 1500	HB101 + pAR08.2	This study
LCUS 1501	HB101 + pAR10	This study
LCUS 1502	HB101 + pAR11	This study
LCUS 1503	HB101 + pAR12	This study
LCUS 1531	NEBexpress + pAR06.2	This study
LCUS 1532	NEBexpress + pAR09.2	This study
LCUS 1533	NEBexpress + pAR18	This study
LCUS 1535	NEBexpress + pAR20	This study
LCUS 1536	NEBexpress + pAR21	This study
LCUS 1543	NEBexpress + pAR23	This study
LCUS 1576	NEBexpress + pAR24	This study
*Clostridioides difficile* strains		
R20291		[62]
R20291 FM2.5		[13]
LCUS 1507	R20291 FM2.5 + pAR05.2	This study
LCUS 1508	R20291 FM2.5 + pAR03	This study
LCUS 1509	R20291 FM2.5 + pAR04.2	This study
LCUS 1510	R20291 FM2.5 + pAR07.2	This study
LCUS 1511	R20291 FM2.5 + pAR08.2	This study
LCUS 1512	R20291 FM2.5 + pAR10	This study
LCUS 1513	R20291 FM2.5 + pAR11	This study
LCUS 1514	R20291 FM2.5 + pAR12	This study
LCUS 1517	R20291 Δ*slpA*	This study
LCUS 1529	R20291 Δ*slpA *+ pRPF233.2	This study
LCUS 1530	R20291 Δ*slpA *+ pAR01	This study
LCUS 1537	R20291 Δ*slpA *+ pAR06.2	This study
LCUS 1538	R20291 Δ*slpA *+ pAR09.2	This study
LCUS 1539	R20291 Δ*slpA *+ pAR18	This study
LCUS 1542	R20291 Δ*slpA *+ pAR21	This study
LCUS 1568	R20291 FM2.5* *+ pAR20	This study
LCUS 1611	R20291 FM2.5* *+ pAR23	This study
LCUS 1612	R20291 FM2.5* *+ pAR24	This study
*Bacteriophages*		
ΦCD38–2	Leicestervirus; siphophage	[13]
ΦCD111	Leicestervirus; siphophage	[13]
ΦCD146	Leicestervirus; siphophage	[11]
ΦCD508	Colneyvirus; myophage	[55]
ΦMMP02	Colneyvirus; myophage	[59]
ΦMMP03	Yongloolinvirus; myophage	[59]
ΦMMP04	Sherbrookevirus; myophage	[59]
*Plasmids*		
pDIA6555	Plasmid backbone for allelic exchange mutant construction using endogenous CRISPR-Cas system	[63]
pRPF233.2	pRPF144:SLCT-4	[13]
pJAK003.2	pRPF144:SLCT-10	[13]
pJAK18.2	pRPF144:SLCT-6	[13]
pJAK19.2	pRPF144:SLCT-8	[13]
pAAM0013.2	pRPF144:SLCT-11	[13]
pRPF144E	Plasmid backbone carrying P_cwp2_ promoter	[64]
pRPF144.gg	pRPF144-BsmBI	This study
pAR01	pRPF144:SLCT-4ΔD2end	This study
pAR03	pRPF144:SLCT-4ΔD2start	This study
pAR04.2	pRPF144:SLCT-4ΔD2middle	This study
pAR05.2	pRPF144:SLCT-10ΔD2	This study
pAR06.2	pRPF144.gg:SLCT-L4H11	This study
pAR07.2	pRPF144:SLCT-L11H4	This study
pAR08.2	pRPF144:SLCT-L10H4	This study
pAR09	pRPF144.gg:SLCT-L4H10	This study
pAR10	pRPF144:SLCT-6ΔD2	This study
pAR11	pRPF144:SLCT-L6H4	This study
pAR12	pRPF144:SLCT-8ΔD2	This study
pAR17	pDIA6555:*slpA* spacer	This study
pAR18	pRPF144.gg:SLCT-L4H6	This study
pAR20	pRPF144.gg:SLCT-L8H4	This study
pAR21	pRPF144.gg:SLCT-L4H8	This study
pAR23	pRPF144.gg:SLCT-H4	This study
pAR24	pRPF144.gg:SLCT-H8	This study

### Bacteriophage amplification and titration

Phage lysates were prepared in TY broth using standard phage amplification protocols, and titers were determined using the soft agar overlay method, as described previously [65]. Phage lysates were filtered through 0.2 µm membranes and stored at 4°C. Phage titers were verified regularly, and stocks contained >10^8^ PFU/mL. All phages used in this work are presented in Table 2.

### Domestication of pRPF144E into pRPF144.gg for Golden Gate assembly

Domestication of pRPF144E vector [66] was accomplished with 3 consecutive cycles of PCR mutagenesis, amplicon phosphorylation and blunt-ended ligation. First, a naturally occurring BsaI site in pRPF144E was destroyed and switched to a SalI site. Secondly, two BsmBI sites were added to the domesticated pRPF144E downstream from the P_cwp2_ promoter by primer extension. Lastly, using PCR mutagenesis and primer extension, two BsaI sites were added upstream of the *catP* gene by modifying SmaI into a BsaI site and adding a full BsaI site via the primer extension method. Linear amplicons were first purified using the QIAGEN QIAquick PCR Purification Kit. Then, the amplicon ends were phosphorylated using 10 U of T4 polynucleotide kinase in a mix of 100 ng of linear DNA in 1X T4 DNA ligase buffer. The reaction was incubated at 37^o^C for 1 hour. Blunt ended ligation was used to circularize the plasmid in a mix of 80 ng of phosphorylated amplicons, 1X T4 DNA ligase Buffer and 400 U of T4 DNA ligase. Ligation reactions were incubated at room temperature for 2 hours and stored at -20^o^C until use.

### Cloning of modified SLCTs in *C. difficile*

Modified SLCT isoforms from SLCT-6, -8, -10, and -11 were subcloned into the pRPF144.gg, a derivative pRPF144E containing two BsmBI restriction sites positioned downstream the constitutive P_cwp2_ promoter, allowing Golden Gate assembly cloning of these genes under P_cwp2_ control [67]. To do this, fragments from genes encoding the different isoforms from the pJAK019, pJAK020, pJAK022, and the pAAM013 plasmids were amplified by PCR. Two BsmBI restriction sites were incorporated at both ends of the amplified fragment to enable Golden Gate assembly with the pRPF144.gg plasmid. The various PCR fragments were combined at a 2:1 ratio with 50 ng of the plasmid backbone in the Golden Gate reaction mix, which contained 0.01 mg bovine serum albumin (BSA), 1X T4 DNA ligase buffer, 5 U of BsmBI-v2, and 200 U of T4 DNA ligase. The reaction was subjected to a thermocycling protocol consisting of 96 cycles of digestion at 42°C for 5 minutes, followed by ligation at 16°C for 5 minutes. This was followed by an enzyme inactivation step at 50°C for 5 minutes and a final ligase inactivation step at 80°C for 10 minutes. Cloning into the pRPF144E was also achieved through Gibson Assembly by using NEB Gibson Assembly Master Mix (E2611). The resulting Golden Gate or Gibson assembly mixture was used to transform *E. coli* NEBexpress, CA434 or HB101 competent cells. Transformants were checked by PCR for plasmid presence. Plasmid DNA was extracted using Macherey Nagel NucleoSpin Plasmid extraction kit or NEB Monarch Plasmid Miniprep Kit and purified using Macherey-Nagel NucleoSpin Gel and PCR Clean-up kit or QIAGEN QIAquick PCR Purification Kit for PCR Cleanup. Clones were then verified by Sanger sequencing at the Université Laval sequencing centre or sent to Integrated DNA technologies. The validated plasmids were conjugated into the *C. difficile* FM2.5 strain or R20291 Δ*slpA* as described below. The list of primers used for this study is summarized in S5 Table.

### Deletion of the *slpA* gene in *C. difficile* strain R20291

The *slpA* gene was deleted from the chromosome of *C. difficile* strain R20291 (NC_013316) by allelic exchange using the endogenous CRISPR-Cas system, as previously described [63]. To construct editing plasmids, pDIA6555 was linearized to remove the original *hfq* targeting spacer, which was then replaced with a spacer sequence targeting *slpA*. Then, 1,200 bp regions flanking the *slpA* gene on each side were PCR-amplified from R20291 genomic DNA and all the fragments were cloned into the pDIA6555 vector by Gibson assembly (Table 2). The resulting construction (pAR17) was transformed into *E. coli* HB101, verified by Sanger sequencing and then conjugated into *C. difficile* strain R20291. Allelic exchange was induced by plating on TY agar supplemented with 500 µg/mL anhydrotetracycline to induce the expression of the CRISPR cassette. Colonies were screened for plasmid loss by parallel streaking on TY plates with or without 15µg/mL thiamphenicol. The deletion of *slpA* was confirmed by colony PCR and whole-genome sequencing using both Illumina and Nanopore technologies.

### Conjugation of plasmid DNA into *C. difficile*

Plasmids carrying the different mutated SLCTs described previously were transferred by conjugation into the FM2.5 mutant strain or R20291 Δ*slpA* as previously described [18]. All manipulations were performed under an anaerobic atmosphere using pre-reduced medium and buffer. Briefly, the different *E. coli* NEBexpress, CA434 or HB101 donor strains containing SLCT plasmids were grown overnight in LB broth containing 25 µg/mL chloramphenicol and 50 µg/mL kanamycin for CA434 and NEBexpress or 25 µg/mL chloramphenicol and 50 µg/mL ampicillin for HB101. Transconjugants were verified by colony PCR for the presence of the plasmid using SLCT-specific primers (S5 Table).

### Glycine extraction of cell surface proteins

We assessed the expression of the SlpA proteins by SDS-PAGE after performing a surface protein extraction of the cultures. The samples were prepared as follows: 10 mL of the *C. difficile* cultures at an optical density of 600 nanometers (OD_600_) of 1 were harvested by centrifugation at 4,000 *g* at 4°C, followed by a washing step with 1X phosphate-buffered saline (PBS). The pellet was resuspended in 200 µL of 0.2 M glycine, pH 2.2, followed by incubation at room temperature for 30 min. Cells were centrifuged again for 5 min at 10,000 *g* at room temperature and then 150 µL of the supernatant were transferred into a new tube and the pH was adjusted to 7.5 using 50 µL of a 2 M Tris-HCl solution. Then, 15 µL of the samples were mixed with 5 µL of 4 X loading buffer (200 mM Tris-HCl, pH 6.8, 400 mM dithiothreitol [DTT], 8% SDS, 0.4% bromophenol blue, and 40% glycerol). Samples were then separated on a 12% denaturing polyacrylamide gel (BioShop) using a Mini- Protean tetra cell apparatus (Bio-Rad, Mississauga, ON, Canada) at a constant amperage of 0.20 mA for 1h. The gels were stained with Coomassie blue. Amino acid sequences of all modified S-layer isoforms are listed in S6 Table.

### Phage susceptibility assays on SLCT-modified complemented strains

Phage susceptibility was assessed by spot assays using a standard soft agar overlay method, as previously described [65]. The FM2.5 and R20291 Δ*slpA* strains carrying *slpA* complementation plasmids were tested. The day before the experiment, a preculture of the complemented strain was inoculated into 5 mL of TY broth supplemented with thiamphenicol (15 µg/mL) and incubated overnight at 37°C under anaerobic conditions. The following day, a fresh 5 mL culture was inoculated with 3% of the overnight preculture, and grown under the same conditions, with thiamphenicol added for plasmid maintenance. The culture’s OD_600_ was then monitored regularly. Meanwhile, soft agarose (0.3%) was prepared and maintained at 55°C. When the bacterial culture reached an OD_600_ of 0.4, 0.67 mL of culture was mixed with 4 mL of soft agarose, 0.1 mL of salt solution (3,8 M MgCl_2_ and 0.1M CaCl_2_) and with antibiotics when necessary (15 µg/mL thiamphenicol). The mixture was poured over square petri dishes containing TY bottom agar (1% agar), supplemented with thiamphenicol. After the top agar solidified, 5 µL of serially diluted phage lysates (initial stocks adjusted at 10^8^ PFU/mL) were spotted directly on top of the soft agarose overlay. Plates were incubated overnight at 37°C in an anaerobic chamber. Zones of lysis and plaque formation in the bacterial lawn revealed productive phage infection.

### Bacteriophage adsorption assays

Phage adsorption assays were carried out as previously described [18] and all manipulations were done in anaerobic conditions. Briefly, *C. difficile* cultures grown overnight in TY broth were used directly for the adsorption assay. For each assay, 0.9 mL of an overnight bacterial culture at an OD_600_ of 1.3-1.4 was mixed with approximately 1 × 10⁵ PFU (plaque forming unit) of the phage of interest in the presence of 10 mM MgCl₂ and 10 mM CaCl₂, in a final volume of 1 mL. The suspensions were incubated for 30 minutes at 37 °C to allow phage adsorption. After incubation, bacterial cells were pelleted by centrifugation at 13,000 *g* for 1 minute. Unabsorbed phages remaining in the supernatant were collected, serially 10-fold diluted and titrated by soft agar overlays as previously described. The percentage of adsorption was calculated as follows: 100 – [(residual titer/ initial titer) × 100]. To increase throughput, the assay was also adapted to a 96-well plate format. The same procedure was followed, using a final volume of 200 µL per well, consisting of 180 µL of overnight bacterial culture and 20 µL of phage suspension containing approximately 5 × 10⁴ PFU. After incubation, bacterial cells were then pelleted by centrifugation at 974 *g* for 3 minutes using a microplate adapter. Supernatants were then titrated using the spot test method on soft agar overlays as described above. All experiments were performed in three technical replicates with a minimum of two biological replicates, and data were presented as the mean adsorption values ± standard deviation (SD) in percentage. Bar plots were generated using R (version 4.3.2) in RStudio.

### Efficiency of plaquing (EOP) assays

EOP assays were performed to compare the phage infection efficiency between the SCLT-4 complemented R20291 Δ*slpA* and R20291 WT strain. Briefly, a series of 10-fold dilutions of each phage lysate (ΦCD146, ΦCD111 and ΦCD38–2) were prepared. Then, 100 μL of the dilutions yielding well-isolated plaques, typically 30–300 PFU per plate, were mixed with a *C. difficile* culture at an OD_600_ of 0.4 in a soft agar overlay to create a bacterial lawn. The average PFU/mL values for each phage on SCLT-4 complemented R20291 Δ*slpA* strain were calculated from a minimum of two biological replicates, each with two technical replicates which were then compared against their titer on the WT strain. The EOP is calculated using the following formula: EOP = Mean PFU on test strain/Mean PFU on WT strain, as described previously [65,68].

### Microplate growth curve assays

A tube with fresh 5 mL broth was inoculated with 3% of an overnight culture and incubated at 37°C under anaerobic conditions until the OD_600_ reached 0.1 to 0.15. Then, a 96-well microplate was prepared by dispensing 200 µL of the bacterial culture into wells in technical triplicates. A negative growth control, consisting of TY medium alone, was also included. The microplate was sealed to maintain anaerobic conditions (Biotechne, Cat. No. DY992) and placed in a preheated plate reader (Epoch 2 Microplate Spectrophotometer, BioTek Instruments, Cat. No. 7172002) at 37°C. Bacterial growth was monitored by measuring the OD_600_ every 10 minutes for 24 hours, with linear shaking for 5 seconds before each measurement. For each bacterial strain, technical triplicates were first averaged to obtain a single value per biological replicate. Subsequently, the mean and standard deviation were calculated across all biological replicates for each condition and time point.

### Data visualization and statistical analysis

Bar plots were generated using R (version 4.3.2) in RStudio. Graphs were created using the ggplot2 package (version 3.5.2) to visualize phage adsorption rates on different *C. difficile* strains. Values were expressed as means ± SD (%), with error bars representing standard deviations. Individual data points were overlaid on the bars. Growth curves graphs were performed using GraphPad Prism software version 10.5.0 (Boston, Massachusetts USA).

### Statistical analysis

All statistical analyses were performed using GraphPad Prism. For each adsorption value comparison involving at least three data points, the distribution of the data was first evaluated using the Shapiro-Wilk normality test. If the data were normally distributed, a Welch’s t-test was performed. If the data did not follow a normal distribution, a Mann–Whitney non-parametric test was applied instead, followed by a t-test with Welch’s correction. Growth curve assays (OD_600_) were averaged from three biological replicates, each measured in technical triplicates. Statistical analysis of global differences between strains was performed using an ordinary two-way ANOVA (factors: strain and time), followed by Tukey’s multiple comparisons test of marginal means. Statistical significance was set at P < 0.05. Statistical significance was defined as P < 0.05 (*), P < 0.01 (**), P < 0.001 (***) and P < 0.0001 (****).

### Protein structure prediction

Structural models of SLCT-4 WT and variants with partial D2 deletion, as well as WT and full D2 deletions in SLCT-6, SLCT-8 and SLCT-10, were generated using the AlphaFold3 server with default settings [43]. For each construct, the high molecular weight (HMW) and low molecular weight (LMW) regions, excluding the signal peptide, were provided as separate chains to improve prediction accuracy of the folded fragments. Among the five models returned by AlphaFold3, the top-ranked structure based on the internal confidence score was selected for further analysis. Structural visualizations and comparisons were performed using PyMOL Molecular Graphics System, Version 2.5.2 (Schrödinger, LLC).

### Bacterial genome sequencing and analysis

Whole bacterial genome DNA was extracted using the Monarch Spin gDNA Extraction Kit (NEB, Cat. No. T3010S). Short-read libraries were prepared with the Ultra II FS DNA Library Prep Kit (NEB) and sequenced on an Aviti system (2x300bp paired-end, Element Biosciences). Long-read sequencing libraries were prepared using the Rapid Barcoding kit (SQK-RBK114.94, Oxford Nanopore Technologies), following the manufacturer’s instructions and sequenced on a PromethION P2 Solo. Basecalling was performed using Dorado with the Super Accuracy model (v4.3.0). The raw reads were then used to reconstruct the genomes using the wf-bacterial-genomes pipeline from Epi2me Labs (Oxford Nanopore Technologies) with default parameters (https://github.com/epi2me-labs/wf-bacterial-genomes). Mapping to the *C. difficile* R20291 reference genome (accession number NC_013316.1) and variant calling were done using BWA and GATK, with annotation via the Variant Effect Predictor (EnsemblBacteria). Missense mutations were mapped to predicted protein domains using InterProScan version 5.75-106.0 (EMBL-EBI) to evaluate their potential functional impact. All library preparation, sequencing, genome assembly and annotation were performed by the RNomics platform (Université de Sherbrooke). Fastq files trimming and variant calling were done by the bio-informatic platform (Université de Sherbrooke).

## Supporting information

S1 Fig Efficiency of plating (EOP) of phages ΦCD146, ΦCD111, and ΦCD38–2 on the complemented strain R20291 △*slpA + *SLCT-4 relative to the WT strain R20291. EOP values were calculated as the ratio of plaque-forming units (PFU) obtained on the tested strain compared to the WT strain. Bars represent the mean log_10_(EOP) from two independent biological replicates, each with two technical replicates. Error bars indicate standard deviation. The table summarizes the mean EOP ± SD for each phage tested.
(S1_Fig.TIF)

S2 Fig Phage susceptibility of R20291 Δ*slpA* expressing SLCT-4 variants with partial D2 deletions. Spot tests on R20291 Δ*slpA* strains complemented with SLCT-4 variants carrying partial D2 deletions (ΔD2 start, ΔD2 middle, and ΔD2 end), which express only the HMW-4 fragment. Serial 10-fold dilutions of the indicated phages (undiluted titers >10⁸ PFU/mL) were spotted onto bacterial lawns. No zones of lysis or plaques were observed. This experiment was performed once.
(S2_Fig.TIF)

S3 Fig AlphaFold3 structural predictions of SLCT-4 variants with partial D2 deletions. Predicted full-length structures of SLCT-4 and its variants with full or partial deletions in the D2 domain, generated using AlphaFold3. Input sequences correspond to mature proteins (signal peptides removed), with LMW and HMW fragments submitted separately. Domains are colored as follows: D1 (yellow), D2 subregion start (blue), middle (green), and end (orange), and LID/HID-HMW (purple). Structural models illustrate the effects of specific D2 subdomain deletions on global protein folding and domain architecture.
(S3_Fig.TIF)

S4 Fig Phage susceptibility of FM2.5 complemented with SLCT-H4 or SLCT-H8. Spot tests on FM2.5 strains expressing SLCT-H4 or SLCT-H8. Serial 10-fold dilutions of the indicated phages (undiluted titers >10⁸ PFU/mL) were spotted onto bacterial lawns. For ΦMMP04, the observed lysis zone on the SLCT-H4-complemented strain is considered to result from a lysis from without phenomenon, as described in the main text. This experiment was performed once.
(S4_Fig.TIF)

S5 Fig Phage susceptibility of strains expressing chimeric SLCT-L6/H4 and SLCT-L4/H6. Spot tests on FM2.5 and R20291 Δ*slpA* strains complemented with SLCT-L6/H4 and SLCT-L4/H6, respectively. Serial 10-fold dilutions of the indicated phages (undiluted titers >10⁸ PFU/mL) were spotted onto bacterial lawns. Infections confirmed as positive are highlighted in red. Zones of lysis observed with certain phages are presumed to result from lysis from without (ΦMMP04) or non-productive interactions (ΦMMP02) as noted in the main text. Experiments were repeated at least twice.
(S5_Fig.TIF)

S6 Fig Phage susceptibility of strains expressing chimeric SLCT-L8/H4 and SLCT-L4/H8. Spot tests on FM2.5 and R20291 Δ*slpA* strains complemented with SLCT-L8/H4 and SLCT-L4/H8, respectively. Serial 10-fold dilutions of the indicated phages (undiluted titers >10⁸ PFU/mL) were spotted onto bacterial lawns. Infections confirmed as positive are highlighted in red.
(S6_Fig.TIF)

S7 Fig Phage susceptibility of strains expressing chimeric SLCT-L10/H4 and SLCT-L4/H10. Spot assays on FM2.5 and R20291 Δ*slpA* strains complemented with SLCT-L10/H4 and SLCT-L4/H10, respectively. Serial 10-fold dilutions of the indicated phages (undiluted titers >10⁸ PFU/mL) were spotted onto bacterial lawns. Zones of lysis observed with certain phages are presumed to result from lysis from without (ΦMMP04) or non-productive interactions (ΦMMP03) as noted in the main text.
(S7_Fig.TIF)

S8 Fig Phage susceptibility of strains expressing chimeric SLCT-L11/H4 and SLCT-L4/H11. Spot assays on FM2.5 and R20291 Δ*slpA* strains complemented with SLCT-L11/H4 and SLCT-L4/H11, respectively. Serial 10-fold dilutions of the indicated phages (undiluted titers >10⁸ PFU/mL) were spotted onto bacterial lawns. Zones of lysis observed with certain phages are presumed to result from lysis from without (ΦMMP04) or non-productive interactions (ΦMMP03) as noted in the main text.
(S8_Fig.TIF)

S1 Table Adsorption data. (S1_Table.XLSX)

S2 Table SNPs detected in R20291 △*slpA.* (S2_Table.XLSX)

S3 Table SNPs detected in FM2.5. (S3_Table.XLSX)

S4 Table SNPs detected in R20291. (S4_Table.XLSX)

S5 Table Primer list. (S5_Table.XLSX)

S6 Table Amino acid sequences of SLCTs used in this study. (S6_Table.XLSX)
